# Village Charities

**Published:** 1890-02-08

**Authors:** 


### Village Charities.

### I.—HARD-AND-FAST RULES OF DISTRIBUTION.

"Many are the troubles of the righteous," says the Psalmist. And certain it is that not the least of his troubles are those that beset him when he is giving, or thinking of giving, his charities. He knows that even charity may be selfish; that he needs to be ever on his guard against the self-indulgence which prompts him to relieve the uncomfortable feelings which the sight of want or privation rouses within him—at the expense, it may be, of the person he would like to help. And so it often comes to pass that he turns to his charities with something very much like a sigh.

But, after all, it is the abuse and not the use of charities against which we have to guard ourselves. "The poor we have always with us;" and while this is so, they must of necessity have a claim on our charity—not on our sentimentality, not on our easy, careless good nature, but on a charity which, though springing from the heart, should be guided by the head, which shall discriminate as well as sympathise, which shall never lose sight of the permanent and moral, in considering the immediate and material, welfare of its objects. At this season of the year, when our hearts naturally seek to share with our less fortunate neighbours some of the comforts which rob King Winter of all his terrors for us, it seems well to think over our charities, that we may make our benevolence less haphazard, more intelligent, and more permanently useful than it now too often is.

With such a large and complex subject before us, we can do no more than touch upon a few of the difficulties connected with it, and suggest one or two principles on which to work. It is only a truism to say that, in a village especially, where everyone knows everyone else's affairs, the distributor of Christmas gifts—coals, blankets, &c.—has often a most thankless task, and almost dreads going out of doors, knowing how he will be assailed with appeals, reproaches, assurances that "if he only knowed, he'd never have given away what he did to a man who drinks away all bis money," or (if he happens to be a clergyman) that "everyone knows that's what Mary Jones come to the Table for." "Ah, those Christmas presents, they gnawed a many!" exclaimed a village woman once; and really, when he sees the angry, jealous faces around him, he is tempted to wonder whether the village would not have been happier and better with no Christmas gifts at all.

Yet he has practically no choice in the matter. These charities have often been left to the village by some worthy, long since deceased; and even where they are provided through collections made year by year among the well-to-do inhabitants—well, it is good for them, and for the poor also, that they should show themselves willing to relieve distress. The only thing to do in such cases is to lay down strict rules for the distribution of the gifts, to let these rules be known to the people, and then to stick to them through thick and thin. Nothing can be worse for the villagers than to know that if they can somehow recommend themselves to the parson, or give him the impression that they are poorer than they are, they may get a share in the charity; nothing can be worse, again, than that they should feel themselves able, by an artful insinuation, or a deliberate mis-statement, to injure a neighbour's chance of the good things going. But let them once understand that the coals, say, will only go to widows—sick people—the aged and infirm—parents with a certain number of children under ten or thirteen (adding to or subtracting from this list, according to the amount of coal to be given), and the difficulties will soon vanish. Far better let a widow in comparatively easy circumstances be on the list, or relieve by a little private gift a poor family who will not come under any of the various heads, than throw the village into a turmoil by what must seem like a special spite or a special mark of favour.

It may be hard sometimes to leave out some family with poverty and privation plainly written in their faces. But we must remember that, unless there are obvious causes for their poverty (causes which would bring them under one of the heads already mentioned), it is probably due to want of thrift idleness, or mismanagement. "I must be cruel only to be kind," is a dictum which, in the very interest of the poor themselves, we cannot afford to lose sight of; and although there may be exceptional cases in which we cannot but relieve extreme and pressing want, however it may have been produced, they should be treated and spoken of *as* exceptional, that no colour may be given to the statement, so readily and so often made, that it seems to pay best to be lazy, self-indulgent, and reckless. In fact, it would be far better to proceed on the opposite plan, if only we can devise some satisfactory mode of doing it, and only help those who are evidently trying to help themselves. Surely they have hit upon the true principle who urge that out-door relief should only be given to such aged persons as can show that they have been "kind to their old age," and made some small provision for it.

By keeping closely to some such hard-and-fast rules as those suggested above, we shall have at least done something to prevent the clergyman—who is generally the alms-distributor in a country parish—from being charged with favouritism, and a desire to get all the benefits for his own flock. It is all-important that his character for fairness and upright dealing should be kept up; if he, the guardian of morals, is supposed to be lax in this respect, what can be expected of his parishioners? Probably the formation of a small committee would be a help to him, by shifting a portion of the burden and responsibility from his shoulders on to those of other people.

For this question is by no means so simple as it at first sight appears. It is easy enough to say, "Oh, of course I shall make no distinction between Churchmen and Dissenters in the matter of charity. They are given help because they need it—not because they hold some special creed." Yes, but how about the Dissenters and their gifts? Do they go upon the same broad principle? Supposing we find—as we may often find—that a village dissenting community is strongly backed up by some well-to-do town, and that its charities are exclusively given to its members? Are such charities as are given through the Church, and by Church people, to take no heed of this? Is a premium to be offered on Dissent? Are Dissenters to have two presents, Church people but one? This is so manifestly unfair, that the special circumstances of such cases must be taken into account; and though Church-going and alms-receiving should be dissociated in the people's minds as far as possible, it may be necessary to put the case simply before them, pointing out the injustice and harm which might ensue if no heed were taken of such things. A committee can better undertake this work than can a clergyman, single-handed; moreover, it will be a great advantage to him, when asked for help on his parochial rounds, to say simply that the committee must be applied to, or that he will lay the case before it, rather than feel, and let the applicant feel, that the decision rests entirely with him.

To sever the time-honoured connection between religion and alms-receiving should be the aim of all who really "consider the poor." It is not many years since in some village churches the contents of the offertory-bag were distributed at the altar-rails when service was over, among such of the poor as were communicants; and it was with a full conviction that she did well to be angry that a cottage-woman, in easy circumstances, lately exclaimed to a clergyman's daughter that "she did think the flock ought to be encouraged; as for herself, she had never had so much as would sit upon a pin's head since she came to the place, and she a Churchwoman, too!"

It is partly the fault of our preachers and teachers that many of the poor are apt to look upon religion as a matter of policy, a mere bargain between man and his Maker—so much worship on the one side, so many benefits on the other. Let us beware of adding to their temptations by letting them discover that the only way to secure benefits from us also is to be, or profess to be, religious. The course of the world's affairs indeed shows a connection between goodness and prosperity, or at any rate happiness, obvious enough to all who have eyes to see; but that is no reason why we should lay undue and unnatural emphasis upon this connection; indeed, by so doing, we run the risk of destroying the very goodness—taking the word in its highest and only true sense —which we would fain foster.

*(To be continued.)*

